# Coronary angiography findings in emergency department chest pain patients undergoing angiography despite hs-cTnT-based early rule-out angiography after hs-cTnT rule-out in ED chest pain

**DOI:** 10.1136/openhrt-2026-004186

**Published:** 2026-07-09

**Authors:** Abdullah Avlac, Ertuğrul Altınbilek, Bedriye Müge Sönmez, Derya Öztürk, İlayda Civelek, Selin Çelik

**Affiliations:** 1 İstanbul Şişli Hamidiye Etfal Eğitim ve Araştırma Hastanesi, Şişli, İstanbul, Turkey; 2 Emergency Medicine, Dokuz Eylul Universitesi Hastanesi, Izmir, Turkey

**Keywords:** Chest Pain, Coronary Angiography, Coronary Stenosis, Biomarkers, Acute Coronary Syndrome

## Abstract

**Background:**

High-sensitivity cardiac troponin (hs-cTn) algorithms are established for early rule-out of acute myocardial infarction, but coronary angiographic (CAG) findings in emergency department (ED) patients undergoing invasive evaluation despite fulfilling rule-out criteria remain insufficiently described.

**Objective:**

To characterise the coronary anatomy and clinical profile of ED patients with chest pain undergoing urgent CAG despite fulfilling hs-cTnT-based early rule-out criteria.

**Methods:**

This retrospective single-centre study included adults with suspected acute coronary syndrome from 1 January 2020 to 1 January 2023. All patients fulfilled the serial European Society of Cardiology 0/1-hour rule-out pathway, defined as baseline hs-cTnT <12 ng/L and a 1-hour absolute change <3 ng/L. Patients undergoing urgent CAG during index hospitalisation comprised the angiographic cohort. Findings were categorised as significant stenosis (≥70% in a major non-left-main epicardial artery or ≥50% in the left main), intermediate stenosis (50%–69% in a non-left-main artery) or non-obstructive coronary artery disease (<50%). Baseline and 1-hour hs-cTnT concentrations, 1-hour and relative 1-hour changes, History, ECG, Age, Risk factors and Troponin (HEART) scores and angiographic findings were compared.

**Results:**

Of 646 patients, 376 underwent urgent CAG. Significant stenosis was found in 109 (29.0%), intermediate stenosis in 220 (58.5%) and non-obstructive disease in 47 (12.5%). Baseline and 1-hour hs-cTnT concentrations and HEART scores differed significantly across angiographic categories, with the highest values in the significant stenosis group and the lowest in the non-obstructive group. The 1-hour and relative 1-hour hs-cTnT changes also differed overall, although significant pairwise differences were limited to the intermediate and non-obstructive groups. Relative hs-cTnT change showed limited discrimination for non-obstructive disease, whereas the HEART score showed moderate discrimination for ≥50% angiographic stenosis.

**Conclusions:**

Among selected ED patients undergoing urgent CAG despite hs-cTnT-based rule-out, intermediate or significant stenosis was common. These findings descriptively characterise coronary anatomy in this cohort but do not establish culprit-lesion status, functional significance, appropriateness of angiography or failure of the hs-cTnT rule-out algorithm.

**WHAT IS ALREADY KNOWN ON THIS TOPIC**

- High-sensitivity cardiac troponin (hs-cTn)-based pathways are well established for the early rule-out of acute myocardial infarction. However, the coronary angiographic findings of clinically selected patients who undergo urgent invasive evaluation despite fulfilling formal hs-cTnT rule-out criteria remain insufficiently characterised.

**WHAT THIS STUDY ADDS**

- Among clinically selected emergency department patients who underwent urgent coronary angiography despite fulfilling the serial European Society of Cardiology 0/1-hour hs-cTnT rule-out pathway, intermediate stenosis or significant stenosis was frequently observed, whereas non-obstructive coronary artery disease accounted for a smaller proportion of angiographic findings.

**HOW THIS STUDY MIGHT AFFECT RESEARCH, PRACTICE OR POLICY**

- These findings provide hypothesis-generating anatomical data on a clinically selected subgroup of emergency department patients who underwent urgent coronary angiography despite hs-cTnT-based rule-out. They may inform prospective studies aimed at identifying which residual clinical features are associated with intermediate or significant coronary stenosis and which patients may warrant further evaluation after rule-out.

## Introduction

 Nontraumatic chest pain (CP) is one of the most common presentations to the emergency department (ED) and a major trigger for the evaluation of possible acute coronary syndrome (ACS). Contemporary diagnostic pathways have improved the early assessment of suspected ACS through the combined use of clinical evaluation, ECG, high-sensitivity cardiac troponin (hs-cTn), and structured risk assessment.^1^,^4^ However, a subset of patients still proceeds to invasive coronary evaluation, and detailed coronary angiography (CAG) findings in this selected group remain less well characterised in existing ED CP studies.^5^

Most contemporary ED pathways use hs-cTn, ECG and structured risk tools to identify patients who may be safely discharged without further in-hospital testing.^1^,^4^ In real-world practice, however, a clinically selected subgroup still proceeds to CAG during the index hospitalisation.^6^ Understanding this subgroup may help address an underexplored gap in the literature regarding the coronary anatomy and clinical profile of patients selected for invasive evaluation after early biomarker-based rule-out.

To our knowledge, no previous ED-based study has specifically characterised the coronary anatomy and detailed clinical profile of patients who undergo urgent CAG despite meeting formal hs-cTn-based early rule-out criteria for acute myocardial infarction (AMI). Importantly, fulfilment of these criteria does not exclude unstable angina or the presence of underlying coronary artery disease.^4 7^ Previous studies have mainly focused on validating the diagnostic performance and safety of accelerated hs-cTn rule-out pathways or on reporting downstream angiography utilisation.^8^,^11^ Better characterisation of this population may provide a clearer understanding of the coronary findings and clinical features encountered in patients selected for invasive evaluation despite early biomarker-based rule-out. Accordingly, this study aimed to characterise the coronary anatomy and associated clinical features of ED patients with CP who underwent urgent CAG despite fulfilling the serial European Society of Cardiology (ESC) 0/1-hour hs-cTnT rule-out pathway.

## Materials and methods

### Study setting

This retrospective single-centre observational study was conducted at the ED of a tertiary care hospital between 1 January 2020 and 1 January 2023.

### Study population

Patients with acute CP and suspected ACS were identified from a broader administrative screening pool of 20 392 ED presentations coded as CP or CP-related complaints during the 3-year study period. This initial screening population did not represent a homogeneous cohort of suspected ACS or a population in which hs-cTnT-based rule-out was systematically applied. In routine ED practice, patients presenting with acute CP were initially evaluated by the treating emergency physician through history-taking, physical examination, assessment of vital signs, and a 12-lead ECG. This initial assessment aimed to rapidly identify life-threatening conditions and to determine whether the presentation was more consistent with a non-cardiac cause, a non-ischaemic cardiac cause, or possible ACS. Consistent with contemporary American Heart Association/American College of Cardiology (AHA/ACC) CP and ESC ACS pathways, cardiac troponin testing was not initiated solely on the basis of a CP-related presentation code. Rather, hs-cTnT testing was performed in patients in whom ACS remained clinically suspected after the initial assessment and exclusion of ST-Elevation Myocardial Infarctio (STEMI). Further evaluation included serial hs-cTnT measurements, clinical risk stratification, imaging when indicated, and cardiology consultation to guide management and disposition.^1 6^

**Figure 1:**
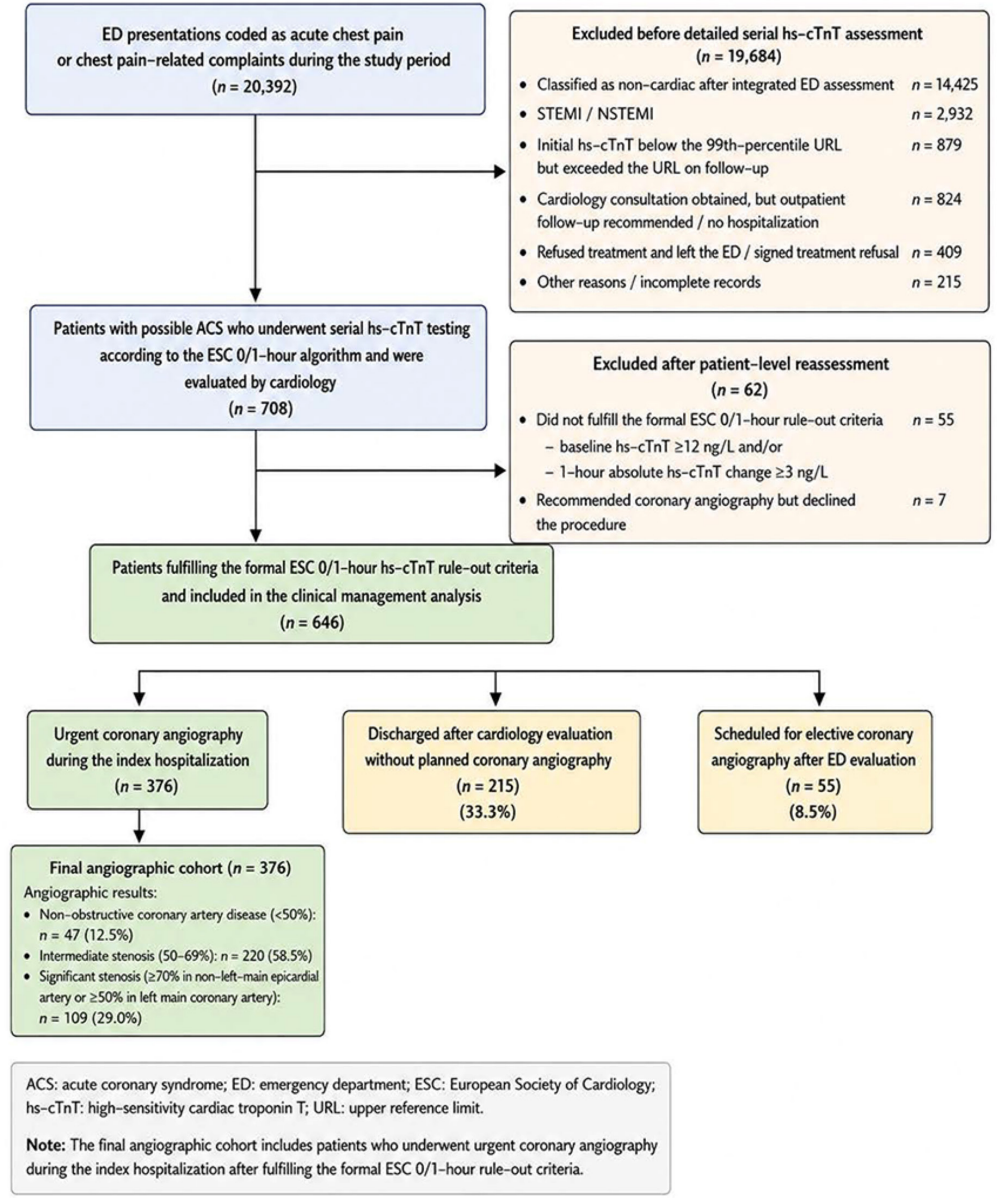
Patient selection and clinical management flow diagram.

Following re-screening of the source database, 708 patients with possible ACS who underwent serial hs-cTnT testing according to the ESC 0/1-hour algorithm and were evaluated by cardiology were identified. After exclusion of 55 patients who did not meet the formal ESC rule-out criteria and 7 patients who declined the recommended CAG, 646 patients constituted the clinical management cohort. Of these, 376 underwent urgent CAG during the index hospitalisation and constituted the final angiographic cohort, whereas 215 were discharged without planned angiography and 55 were scheduled for elective CAG (figure 1).

Eligibility for the clinical management cohort required age ≥18 years; presentation to the ED with acute CP and clinical suspicion of ACS; standardised hs-cTnT measurements at presentation and at 1 hour; fulfilment of the assay-specific ESC 0/1-hour rule-out criteria; cardiology evaluation and availability of the relevant clinical and laboratory records. For the rule-out pathway used in this study, the baseline hs-cTnT concentration was required to be <12 ng/L, with a 1-hour absolute change of <3 ng/L. Patients were excluded if they were aged <18 years, had STEMI on the initial ECG, did not undergo standardised 0/1-hour hs-cTnT sampling, had a baseline hs-cTnT concentration ≥12 ng/L or a 1-hour absolute change ≥3 ng/L, declined the recommended CAG, or had incomplete clinical or laboratory records. The primary angiographic cohort comprised eligible patients who underwent urgent CAG during the index hospitalisation. Patients discharged without planned angiography or scheduled for elective CAG were retained in the clinical management pathway analysis but were excluded from the primary angiographic outcome analysis.

CP was defined in accordance with the 2021 AHA/ACC Chest Pain Guideline as acute CP or chest discomfort in patients with clinically suspected ACS. After STEMI was excluded on the initial ECG, with cardiology consultation obtained when ECG findings were equivocal or suggestive of ischaemia, the History, ECG, Age, Risk factors, and Troponin (HEART) score was calculated using information documented during the initial ED assessment to support structured clinical risk stratification.¹

### Clinical assessment, hs-cTnT testing and CAG

No study-mandated intervention was applied. CAG was performed during the same hospital admission, either emergently or as part of an inpatient evaluation, at the discretion of the treating clinicians and in consultation with cardiology specialists. The decision to proceed to CAG was based on the overall clinical assessment, including symptoms, ECG findings, cardiovascular risk profile and serial hs-cTnT results, rather than predefined study criteria. However, the specific patient-level indication for urgent CAG was not recorded in a standardised data field. Therefore, the individual reasons for invasive evaluation could not be reliably classified retrospectively. hs-cTnT concentrations were measured at presentation and 1 hour later using the Elecsys Troponin T hs assay (Roche Diagnostics GmbH, Mannheim, Germany; REF 09315322190) on a cobas e 601 analyser. Sampling times were defined by blood draw time rather than laboratory reporting time. The assay had a manufacturer-reported limit of detection of 5 ng/L and an overall 99th-percentile upper reference limit of 14 ng/L.^12^ According to the assay-specific ESC 0/1-hour algorithm, rule-out was defined as either a baseline hs-cTnT concentration <5 ng/L in patients presenting more than 3 hours after symptom onset or a baseline concentration <12 ng/L combined with a 1-hour absolute change <3 ng/L, whereas rule-in was defined as a baseline hs-cTnT concentration ≥52 ng/L or a 1-hour absolute change ≥5 ng/L; patients meeting neither the rule-out nor the rule-in criteria were assigned to the observation zone.^6^ Eligibility was restricted to the serial rule-out pathway, which was fulfilled by all 646 patients in the clinical management cohort. Because the symptom onset-to-sampling interval was not consistently documented in this retrospective study, the single-sample pathway was not used to determine eligibility. The absolute 1-hour change used to determine eligibility was calculated as |hs-cTnT at 1 hour−hs-cTnT at presentation|. For subsequent analyses, the 1-hour change was calculated as hs-cTnT at 1 hour−hs-cTnT at presentation. The relative 1-hour change was calculated as [(hs-cTnT at 1 hour−hs-cTnT at presentation)/hs-cTnT at presentation]×100. Accordingly, negative values for the 1-hour or relative change indicate a decrease in hs-cTnT over the 1-hour interval and do not represent failure to meet the eligibility criterion.

### Outcomes

The primary outcome was angiographic stenosis severity among patients who underwent urgent CAG. For the purposes of this study, CAG findings were categorised according to visually estimated diameter stenosis as significant stenosis (≥70% in a major non-left-main epicardial coronary artery or ≥50% in the left main coronary artery; group 1), intermediate stenosis (50%–69% in a major non-left-main epicardial coronary artery; group 2) and non-obstructive coronary artery disease (<50%; group 3).^1 13 14^ Because fractional flow reserve (FFR), instantaneous wave-free ratio (iFR), intravascular ultrasound (IVUS), optical coherence tomography, stress testing and formal lesion-specific clinical adjudication were not systematically available, these categories were based on anatomical appearance and should not be interpreted as establishing functional or ischaemic significance.^14^

Secondary outcomes included differences in baseline characteristics across the three clinical management pathways; associations of hs-cTnT levels, HEART score components and sex with angiographic outcomes; and the discriminative performance of the relative change in hs-cTnT and the HEART score. Demographic, clinical, laboratory, management and angiographic data were extracted from hospital records using predesigned data collection forms.

### Statistical analysis

Statistical analyses were performed using SPSS V.25.0 (IBM). Continuous variables were assessed for distribution by visual inspection of histograms and formal normality testing. Normally distributed variables are presented as mean±SD, whereas non-normally distributed variables are reported as median (IQR). Categorical variables are presented as frequencies and percentages. Comparisons between two independent groups were performed using the independent-samples t-test or Mann-Whitney U test, as appropriate. Comparisons among the three angiographic outcome groups were performed using the Kruskal-Wallis test for continuous or ordinal variables. When the overall test was statistically significant, pairwise post hoc comparisons were performed with a Bonferroni adjustment. Baseline characteristics were also compared across the three clinical management pathways using the Kruskal-Wallis test for continuous or ordinal variables and the Pearson χ^2^ test for sex. When the overall between-group difference was statistically significant, pairwise post hoc comparisons were performed with a Bonferroni adjustment. Receiver operating characteristic (ROC) curve analysis was used to evaluate the discriminatory performance of relative 1-hour hs-cTnT change for identifying non-obstructive coronary artery disease (group 3 vs groups 1 and 2), of the HEART score for identifying angiographic stenosis ≥50% (groups 1 and 2 vs group 3), and of baseline hs-cTnT, 1-hour hs-cTnT, the 1-hour hs-cTnT change, the relative 1-hour hs-cTnT change and the HEART score for identifying significant coronary stenosis (group 1 vs groups 2 and 3). Optimal cut-off values were determined using the Youden index. A two-sided p<0.05 was considered statistically significant.

## Results

### Study population and clinical management pathways

The urgent CAG cohort comprised 376 patients, including 249 men (66.2%) and 127 women (33.8%), with a median age of 61 years (range, 31–88 years). Among patients undergoing urgent CAG, the distribution of angiographic outcomes did not differ significantly between women and men (p=0.581). Age and sex distributions differed significantly among the three clinical management groups (both p<0.001). Patients in the discharged group were younger than those in both the elective and urgent CAG groups, whereas age did not differ significantly between the elective and urgent CAG groups after Bonferroni adjustment. Women constituted approximately half of the discharged and elective CAG groups, but only one-third of the urgent CAG group. Baseline hs-cTnT concentrations were comparable across the three management pathways (p=0.863). Although an overall difference was observed in 1-hour hs-cTnT concentrations (p=0.034), none of the pairwise comparisons remained significant after Bonferroni adjustment. In contrast, both the 1-hour change and the relative 1-hour change in hs-cTnT differed significantly among the groups (both p<0.001). Both change measures were lower in the urgent CAG group than in the discharged and elective CAG groups, whereas no significant difference was observed between the discharged and elective CAG groups (table 1).

**Table 1:** Baseline clinical and laboratory characteristics according to the clinical management pathways

Parameter	Discharged without planned CAG (n=215)	Elective CAG (n=55)	Urgent CAG (n=376)	P value
Age, years	53.00 (47.00–59.00)	61.00 (57.00–64.00)	61.00 (50.00–68.00)	<0.001
Female sex, n (%)	105 (48.8)	29 (52.7)	127 (33.8)	<0.001
Male sex, n (%)	110 (51.2)	26 (47.3)	249 (66.2)	
hs-cTnT at 0 hours, ng/L	6.40 (4.21–8.93)	6.62 (4.59–8.69)	6.38 (4.54–8.54)	0.863
hs-cTnT at 1 hour, ng/L	7.36 (5.22–9.31)	7.33 (5.60–8.96)	6.54 (5.01–8.72)	0.034
1-hour change in hs-cTnT, ng/L	0.58 (0.26–1.01)	0.52 (0.20–1.00)	0.22 (−0.29–0.92)	<0.001
Relative 1-hour change in hs-cTnT, %	10.03 (3.21–18.89)	7.38 (3.59–21.03)	3.73 (−4.38–14.89)	<0.001
HEART score	2.00 (2.00–3.00)	3.00 (3.00–4.00)	4.00 (3.00–5.00)	<0.001
HEART history component	1.00 (1.00–1.00)	1.00 (1.00–1.00)	1.00 (1.00–1.00)	<0.001
HEART ECG component	0.00 (0.00–1.00)	1.00 (0.00–1.00)	0.00 (0.00–1.00)	<0.001
HEART age component	1.00 (1.00–1.00)	1.00 (1.00–1.00)	1.00 (1.00–2.00)	<0.001
HEART risk-factor component	0.00 (0.00–1.00)	1.00 (0.00–1.00)	1.00 (1.00–1.00)	<0.001

Data are presented as median (IQR) or number (%), as appropriate. Comparisons among the three clinical management groups were performed using the Kruskal-Wallis test for continuous or ordinal variables and the Pearson χ2 test for sex. The HEART troponin component was constant across all patients and was therefore not presented.

CAG, coronary angiography; HEART, History, ECG, Age, Risk factors and Troponin; hs-cTnT, hs-cTnT high-sensitivity cardiac troponin .

HEART scores also differed significantly across the management pathways (p<0.001). The discharged group had lower HEART scores than both the elective and urgent CAG groups, while the difference between the elective and urgent CAG groups was not significant after adjustment. Significant overall differences were also observed in all individual HEART components (all p<0.001). Pairwise analyses showed that the history component differed between the urgent CAG group and each of the other groups, the ECG component differed between the elective CAG group and both the discharged and urgent CAG groups, and the age component differed only between the discharged and urgent CAG groups. The risk-factor component differed significantly across all pairwise comparisons. Bonferroni-adjusted pairwise results are provided in online supplemental table S1.

### CAG outcomes and hs-cTnT parameters

Among the 376 patients who underwent urgent CAG, 109 (29.0%) had significant coronary stenosis (group 1), 220 (58.5%) had intermediate coronary stenosis (group 2) and 47 (12.5%) had non-obstructive coronary artery disease (group 3). hs-cTnT parameters according to angiographic outcome are presented in table 2. Both baseline and 1-hour hs-cTnT concentrations differed significantly among the three groups (both p<0.001), and all pairwise comparisons remained significant after Bonferroni adjustment. The 1-hour and relative 1-hour changes in hs-cTnT also differed significantly among the groups (p=0.022 and p=0.004, respectively); for both variables, the only significant pairwise difference was between the intermediate stenosis and non-obstructive coronary artery disease groups (groups 2 and 3). Detailed post hoc comparisons are presented in online supplemental table S2; in addition, intervention-site information, available for 93 of the 109 patients with significant stenosis, is summarised in online supplemental table S5.

**Table 2:** hs-cTnT parameters according to angiographic outcome among patients undergoing urgent coronary angiography

Parameter	Group 1 Significant stenosis (n=109)	Group 2 Intermediate stenosis (n=220)	Group 3 Non-obstructive coronary artery disease (n=47)	P value
hs-cTnT at 0 hours, ng/L	7.70 (5.74–9.15)	6.30 (4.64–8.11)	4.40 (3.30–6.02)	<0.001
hs-cTnT at 1 hour, ng/L	7.64 (6.33–9.64)	6.30 (4.89–8.23)	4.69 (3.97–6.53)	<0.001
1-hour change in hs-cTnT, ng/L	0.31 (−0.48–0.96)	0.14 (−0.36–0.71)	0.61 (−0.05–1.00)	0.022
Relative change in hs-cTnT, %	5.56 (−5.29–14.53)	2.53 (−5.07–11.62)	13.90 (−1.33–21.99)	0.004

Data are presented as median (IQR). Comparisons among the three angiographic outcome groups were performed using the Kruskal-Wallis test. For hs-cTnT at 0 and 1 hour, all pairwise comparisons remained significant after Bonferroni adjustment. For the 1-hour and relative hs-cTnT changes, only the comparison between group 2 and group 3 remained significant after adjustment.

hs-cTnT, high-sensitivity cardiac troponin T.

### HEART score according to angiographic findings

The HEART score and its components according to angiographic outcome are presented in table 3. The median HEART score was significantly higher in the significant stenosis group than in the intermediate stenosis and non-obstructive coronary artery disease groups (5 (IQR, 4–5) vs 4 (IQR, 3–5) and 2 (IQR, 2–3), respectively; p<0.001). All pairwise comparisons remained significant after Bonferroni adjustment (all adjusted p-values<0.001). Significant overall differences were also observed in the history (p<0.001), ECG (p=0.005), age (p<0.001) and risk-factor components (p<0.001). The history component was higher in the significant stenosis group than in both the intermediate stenosis and non-obstructive coronary artery disease groups. The ECG component differed only between the significant stenosis and non-obstructive coronary artery disease groups. The age component was higher in both the significant and intermediate stenosis groups than in the non-obstructive coronary artery disease group, whereas the risk-factor component differed significantly across all pairwise comparisons. Detailed Bonferroni-adjusted comparisons are presented in online supplemental table S3. The distribution of angiographic outcomes did not differ significantly between women and men (p=0.581).

**Table 3:** HEART score, its components and sex according to angiographic outcome among patients undergoing urgent coronary angiography

Parameter	Group 1 Significant stenosis (n=109)	Group 2 Intermediate stenosis (n=220)	Group 3 Non-obstructive coronary artery disease (n=47)	P value
HEART score	5.00 (4.00–5.00)	4.00 (3.00–5.00)	2.00 (2.00–3.00)	<0.001
History component	1.00 (1.00–2.00)	1.00 (1.00–1.00)	1.00 (1.00–1.00)	<0.001
ECG component	0.00 (0.00–1.00)	0.00 (0.00–1.00)	0.00 (0.00–0.00)	0.005
Age component	1.00 (1.00–2.00)	1.00 (1.00–2.00)	1.00 (0.00–1.00)	<0.001
Risk-factor component	1.00 (1.00–2.00)	1.00 (1.00–1.00)	1.00 (0.00–1.00)	<0.001
Sex, n (%)				0.581
Female	33 (30.3)	76 (34.5)	18 (38.3)	
Male	76 (69.7)	144 (65.5)	29 (61.7)	

Data are presented as median (IQR) or number (%), as appropriate. Comparisons among the three angiographic outcome groups were performed using the Kruskal-Wallis test for the HEART score and its components and the Pearson χ2 test for sex. The HEART troponin component was constant across all patients and was therefore not presented. Bonferroni-adjusted pairwise comparisons are provided in online supplemental table S3. Group 1: significant coronary stenosis requiring intervention; Group 2: intermediate coronary stenosis managed medically; Group 3: normal coronary arteries.

HEART, History, ECG, Age, Risk factors and Troponin.

### ROC analyses

**Figure 2:**
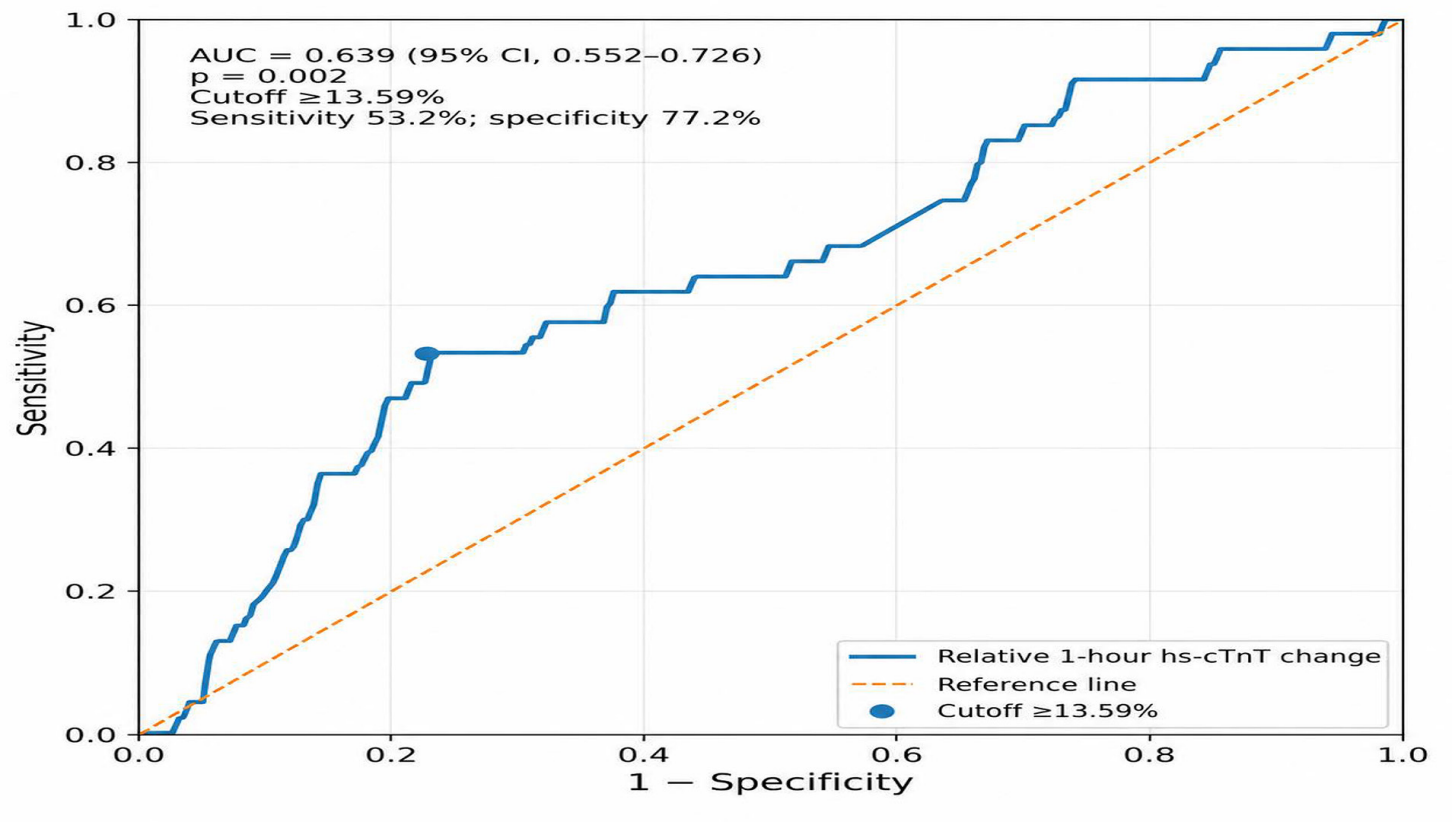
ROC analysis of relative hs-cTnT change for identifying non-obstructive coronary artery disease. hs-cTnT, high-sensitivity cardiac troponin T; ROC, receiver operating characteristic; AUC: area under the curve

**Figure 3:**
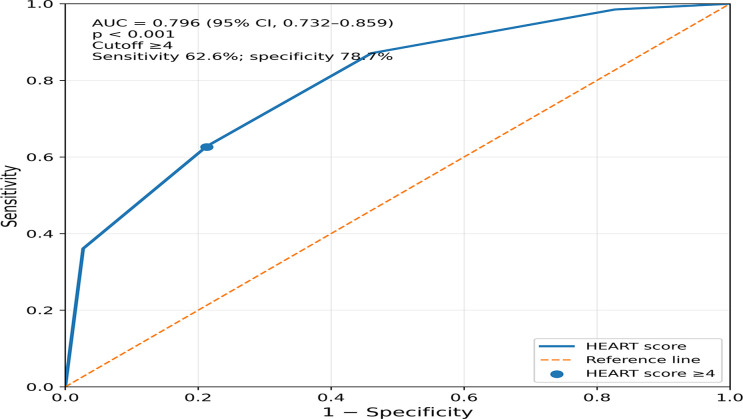
ROC analysis of the HEART score for identifying angiographic stenosis ≥50%. HEART, History, ECG, Age, Risk factors and Troponin; ROC, receiver operating characteristic.

Relative 1-hour hs-cTnT change demonstrated statistically significant but limited discrimination for identifying non-obstructive coronary artery disease (group 3 vs groups 1 and 2), with an Area under the curve (AUC) of 0.639 (95% CI 0.552 to 0.726; p=0.002). The optimal cut-off value, determined using the Youden index, was ≥13.59%, yielding a sensitivity of 53.2% and a specificity of 77.2% (figure 2). The HEART score demonstrated an AUC of 0.796 (95% CI 0.732 to 0.859; p<0.001) for identifying angiographic stenosis ≥50% (groups 1 and 2 vs group 3). The optimal cut-off value was ≥4, with a sensitivity of 62.6% and a specificity of 78.7%. A lower threshold of ≥3 increased sensitivity to 87.5% and corresponding specificity to 53.2% (figure 3).

Additional ROC analyses showed that baseline and 1-hour hs-cTnT concentrations and the HEART score had statistically significant but only modest discrimination for significant coronary stenosis, whereas the 1-hour and relative 1-hour hs-cTnT changes were not discriminatory. The HEART score showed the highest AUC (0.697), and a cut-off of ≥4 yielded 78.9% sensitivity and 51.3% specificity (online supplemental table S4 and online supplemental figure S1).

## Discussion

The principal finding of this study is that intermediate or significant coronary stenosis was common among a clinically selected ED-based subgroup of patients with CP who underwent urgent CAG despite fulfilment of hs-cTnT-based early rule-out criteria. Non-obstructive coronary artery disease was observed in only a small proportion of patients, whereas intermediate and significant stenosis accounted for most angiographic findings. Patients meeting early rule-out criteria are generally considered to have a low probability of AMI; however, these algorithms are not designed to exclude underlying coronary artery disease.^11 15^ These findings therefore describe the coronary anatomy of patients selected for invasive evaluation after early rule-out but do not indicate failure of the hs-cTnT algorithm or establish the clinical relevance of the detected lesions. Previous studies have focused mainly on diagnostic algorithm performance and overall angiography utilisation,^16 17^ leaving the coronary anatomical profile of this subgroup incompletely characterised. Patients selected for urgent CAG were older, more frequently male, and had higher HEART scores than those discharged without urgent angiography, whereas baseline hs-cTnT concentrations were similar across management pathways. These findings suggest that the decision to proceed with urgent invasive evaluation was associated with the integrated clinical risk profile rather than the baseline hs-cTnT concentration alone.

Sex-related differences were evident in clinical management but not in angiographic outcomes. Women were less frequently represented among patients undergoing urgent CAG, although the distribution of significant stenosis, intermediate stenosis and non-obstructive coronary artery disease within the urgent-CAG cohort did not differ by sex. This discrepancy may reflect differences in clinical presentation, perceived risk or referral practices rather than a lower angiographic disease burden among women selected for invasive evaluation. Previous evidence indicating that measurable hs-cTn concentrations within sex-specific reference intervals may retain prognostic importance provides additional context for these findings.^18^ Overall, these findings indicate that sex distribution differed across downstream clinical management pathways, despite comparable angiographic outcome distributions among women and men selected for urgent CAG.

Although single-sample and 0/3-hour hs-cTn strategies are not directly comparable with the assay-specific ESC 0/1-hour algorithm used in the present study, available evidence indicates that very low or sub-99th-percentile hs-cTn concentrations provide high, but not perfect, diagnostic performance for AMI. Importantly, these approaches are designed to rule out AMI rather than the entire spectrum of ACS. Thus, unstable angina may remain despite low or even undetectable hs-cTn concentrations, as reflected by the lower negative predictive value reported for Non–ST-Elevation Myocardial Infarction (NSTE-ACS compared with NSTEMI).^19^,^21^ Consistent with this distinction, negative or very low troponin concentrations do not necessarily exclude clinically relevant coronary artery disease. In troponin-negative patients with atypical CP, Coronary Computed Tomography Angiography (CCTA) identified >50% stenosis in 24.4% of cases, particularly among patients aged ≥50 years and those with intermediate-to-high Framingham risk scores.^22^ Similarly, a Rapid Acute Coronary Syndrome Exclusion using high-sensitivity cardiac I Troponin (RACE-IT) subanalysis showed that 77% of patients diagnosed with unstable angina despite low hs-cTnI concentrations had obstructive disease on CAG.^23^ Accordingly, hs-cTn results should be interpreted in conjunction with symptoms, ECG findings, pretest probability and the overall clinical assessment. These observations provide important context for our findings and demonstrate why selected patients may undergo invasive evaluation despite biomarker-based early rule-out. Our study extends this literature by characterising an ED-based cohort undergoing invasive CAG after hs-cTnT-based early rule-out, in whom intermediate or significant angiographic stenosis was likewise common.

The limited between-group differences observed in the 1-hour and relative 1-hour hs-cTnT changes across angiographic categories are not unexpected. hs-cTn-based algorithms were developed to support the early identification or exclusion of acute myocardial injury and infarction, rather than to define the presence, extent or anatomical severity of epicardial coronary artery disease.^4 24 25^ From this perspective, the absence of a clear gradient in 1-hour hs-cTnT change between angiographic groups should not be interpreted as paradoxical. Rather, it underscores that short-interval troponin kinetics may have limited value in characterising coronary anatomy in a selected cohort that proceeds to invasive evaluation after early rule-out. By contrast, the higher baseline and 1-hour hs-cTnT concentrations observed in patients with more severe angiographic findings may be better understood as contextual clinical signals within this population than as direct markers of stenosis burden.

The HEART score also remained informative within this selected cohort. Patients with significant coronary stenosis had the highest HEART scores, whereas those with non-obstructive coronary artery disease had the lowest, and the score showed moderate discrimination for non-obstructive coronary anatomy. This pattern suggests that, even after hs-cTnT-based early rule-out, the overall clinical profile captured by the HEART score continues to reflect the likelihood of underlying angiographic disease. Importantly, because all patients fulfilled early rule-out criteria, the discriminatory contribution of the score in this setting was driven primarily by its non-troponin components, particularly history, age and risk factor burden, rather than by biomarker elevation itself. In this respect, our findings are consistent with the broader role of the HEART score as a structured clinical risk assessment tool rather than a direct surrogate for coronary anatomy.^326^,^28^ They also align with previous work suggesting that combining troponin results with clinical risk assessment may improve patient stratification in selected presentations, even though very low troponin thresholds may reduce the incremental value of risk scores for rule-out purposes alone.^6 15^

This study has several limitations. First, it was retrospective and single-centre, which limits generalisability and introduces the possibility of unmeasured confounding. Second, although the clinical management analysis included patients across discharge, elective CAG and urgent CAG pathways, the primary angiographic analysis remained restricted to patients selected for urgent CAG. This population was therefore subject to substantial selection bias and confounding by indication, because the decision to perform urgent CAG was based on real-world clinical judgement rather than predefined study criteria. As coronary anatomy was unavailable for patients who did not undergo urgent CAG, the prevalence of coronary artery disease in the overall hs-cTnT rule-out population could not be determined, and the angiographic findings cannot be generalised beyond this selected cohort. Third, the specific clinical indications for urgent CAG were not recorded in a standardised manner. Detailed information regarding the typicality, persistence or recurrence of CP; known coronary artery disease; dynamic ischaemic ECG changes; haemodynamic instability; signs of heart failure; and other high-risk clinical features was not consistently available. Consequently, the individual reasons for proceeding to invasive evaluation could not be reliably classified, and the observed differences across management pathways should be interpreted as group-level associations rather than documented indications for CAG. Fourth, the study focused on angiographic anatomy. The observed coronary stenoses could not be definitively linked to the presenting CP. Because formal culprit-lesion adjudication, intracoronary imaging, physiological lesion assessment and systematic adjudication of unstable angina were not available, the study could not reliably distinguish incidental chronic bystander coronary disease from culprit coronary pathology underlying troponin-negative ACS. In particular, FFR, iFR, IVUS, OCT and stress testing were not systematically available for intermediate lesions; therefore, their haemodynamic or ischaemic significance could not be determined. Although procedural or referral information was available for 93 of the 109 patients with significant stenosis, complete procedural data, standardised culprit-lesion adjudication and final discharge diagnoses were not systematically available. The study also did not evaluate whether the angiographic findings translated into clinically meaningful outcomes, including discharge diagnoses, major adverse cardiovascular events or mortality at 6 months or 1 year, because longitudinal follow-up data were not systematically available. Therefore, the clinical implications of the observed angiographic findings should be interpreted with caution

## Conclusions

In this clinically selected ED-based cohort of patients who underwent urgent CAG despite fulfilling hs-cTnT-based early rule-out criteria, intermediate or significant angiographic stenosis was common, whereas non-obstructive coronary artery disease accounted for a minority of findings. These results provide a descriptive characterisation of coronary anatomy in patients selected for invasive evaluation. The study does not determine whether the rule-out algorithm failed, whether angiography was appropriate, or whether the detected lesions were responsible for the presenting symptoms. Further studies incorporating standardised clinical adjudication, functional lesion assessment and longitudinal outcomes are needed to determine the clinical significance of these findings.

## Supplementary material

10.1136/openhrt-2026-004186 online supplemental figure 1

10.1136/openhrt-2026-004186 online supplemental table 1

10.1136/openhrt-2026-004186 online supplemental table 2

10.1136/openhrt-2026-004186 online supplemental table 3

10.1136/openhrt-2026-004186 online supplemental table 4

10.1136/openhrt-2026-004186 online supplemental table 5

## Data Availability

Data are available on reasonable request.

## References

[1] Gulati M, Levy PD, Mukherjee D (2021). 2021 AHA/ACC/ASE/CHEST/SAEM/SCCT/SCMR Guideline for the Evaluation and Diagnosis of Chest Pain: A Report of the American College of Cardiology/American Heart Association Joint Committee on Clinical Practice Guidelines. Circulation.

[2] Kontos MC, de Lemos JA, Deitelzweig SB (2022). 2022 ACC Expert Consensus Decision Pathway on the Evaluation and Disposition of Acute Chest Pain in the Emergency Department. J Am Coll Cardiol.

[3] Aung SSM, Roongsritong C (2022). A Closer Look at the HEART Score. Cardiol Res.

[4] Byrne RA, Rossello X, Coughlan JJ (2024). 2023 ESC Guidelines for the management of acute coronary syndromes. Eur Heart J Acute Cardiovasc Care.

[5] Sanfilippo FM, Murray K, Hillis GS (2023). Determinants and Outcomes of Invasive Coronary Angiography in Unselected Patients Presenting With Chest Pain to Emergency Departments in Western Australian Teaching Hospitals. Heart Lung Circ.

[6] Jones J, Hughes E, Dobson R (2025). Risk stratification of acute chest pain in patients with high-sensitivity troponin T below the 99th percentile: a long-term cohort study. J Am Heart Assoc.

[7] Twerenbold R, Boeddinghaus J, Nestelberger T (2023). ESC 0/1h-algorithm using the updated, biotin-resistant high-sensitivity cardiac troponin T assay. Eur Heart J.

[8] Odqvist M, Bandstein N, Tygesen H (2023). Outcomes in patients with chest pain in emergency departments using high-sensitivity versus conventional troponins. Scand Cardiovasc J.

[9] Mark DG, Huang J, Lee KK (2025). Validation of a 0-/2-Hour High-Sensitivity Cardiac Troponin Algorithm for Suspected Acute Coronary Syndrome in the Emergency Department. J Am Heart Assoc.

[10] Johannessen TR, Ruud SE, Larstorp ACK (2025). Rapid rule-out of acute myocardial infarction using the 0/1-hour algorithm for cardiac troponins in emergency primary care: the OUT-ACS implementation study. *BMC Prim Care*.

[11] Mokhtari A, Forberg JL, Sandgren J (2024). Effectiveness and Safety of the ESC-TROP (European Society of Cardiology 0h/1h Troponin Rule-Out Protocol) Trial. J Am Heart Assoc.

[12] Roche Diagnostics GmbH (2022). Elecsys troponin T hs: package insert, ref 09315322190/09315322500. version 2.0.

[13] Lawton JS, Tamis-Holland JE, Bangalore S (2022). 2021 ACC/AHA/SCAI Guideline for Coronary Artery Revascularization: A Report of the American College of Cardiology/American Heart Association Joint Committee on Clinical Practice Guidelines. Circulation.

[14] Tamis-Holland JE, Jneid H, Reynolds HR (2019). Contemporary Diagnosis and Management of Patients With Myocardial Infarction in the Absence of Obstructive Coronary Artery Disease: A Scientific Statement From the American Heart Association. Circulation.

[15] Andruchow JE, Boyne T, Innes G (2019). Low High-Sensitivity Troponin Thresholds Identify Low-Risk Patients With Chest Pain Unlikely to Benefit From Further Risk Stratification. *CJC Open*.

[16] Cyon L, Kadesjö E, Edgren G (2024). Long-term prognosis of low high-sensitivity cardiac troponin T in the emergency department compared with the general population. Heart.

[17] Ashburn NP, Snavely AC, Allen BR (2024). Performance of the European Society of Cardiology 0/1-hour algorithm with high-sensitivity cardiac troponin T at 90 days among patients with known coronary artery disease. Am J Emerg Med.

[18] Sandoval Y, Smith SW, Sexter A (2019). Clinical Features and Outcomes of Emergency Department Patients With High-Sensitivity Cardiac Troponin I Concentrations Within Sex-Specific Reference Intervals. Circulation.

[19] Pickering JW, Than MP, Cullen L (2017). Rapid Rule-out of Acute Myocardial Infarction With a Single High-Sensitivity Cardiac Troponin T Measurement Below the Limit of Detection: A Collaborative Meta-analysis. Ann Intern Med.

[20] Sandoval Y, Smith SW, Thordsen SE (2017). Diagnostic Performance of High Sensitivity Compared with Contemporary Cardiac Troponin I for the Diagnosis of Acute Myocardial Infarction. Clin Chem.

[21] Thelin J, Melander O, Öhlin B (2015). Early rule-out of acute coronary syndrome using undetectable levels of high sensitivity troponin T. Eur Heart J Acute Cardiovasc Care.

[22] Westra J, Mohammad MA, Jernberg T (2025). Model development for the prediction of epicardial coronary artery disease in patients presenting with suspected acute coronary syndrome: results from the SWEDEHEART registry. Eur Heart J.

[23] Wattanaseth T, Suvannamai V, Lungkorn N (2025). Coronary artery features in the cases of atypical chest pain with a troponin-negative result. *ASEAN J Radiol*.

[24] Fadel R, Miller J, Cook B Incidence and outcomes of unstable angina in patients with low high-sensitivity cardiac troponin I values: a substudy of the RACE-IT trial.

[25] Wright CX, Wright DS, Hu J-R (2024). High-Sensitivity Troponin: Finding a Meaningful Delta. J Cardiovasc Dev Dis.

[26] Lippi G, Cervellin G, Sanchis-Gomar F (2018). Critical appraisal of using relative or absolute cardiac troponin changes for diagnosing AMI. J Lab Precis Med.

[27] Johannessen TR, Atar D, Vallersnes OM (2021). Comparison of a single high-sensitivity cardiac troponin T measurement with the HEART score for rapid rule-out of acute myocardial infarction in a primary care emergency setting: a cohort study. BMJ Open.

[28] Mahler SA, Riley RF, Hiestand BC (2015). The HEART Pathway randomized trial: identifying emergency department patients with acute chest pain for early discharge. Circ Cardiovasc Qual Outcomes.

