# Supplementary figures and images for: Coronary angiography findings in emergency department chest pain patients undergoing angiography despite hs-cTnT-based early rule-out angiography after hs-cTnT rule-out in ED chest pain
Source: Open Heart. 2026 Jul 9;13(2):e004186. doi: 10.1136/openhrt-2026-004186 (PMC13358279; doi:10.1136/openhrt-2026-004186)

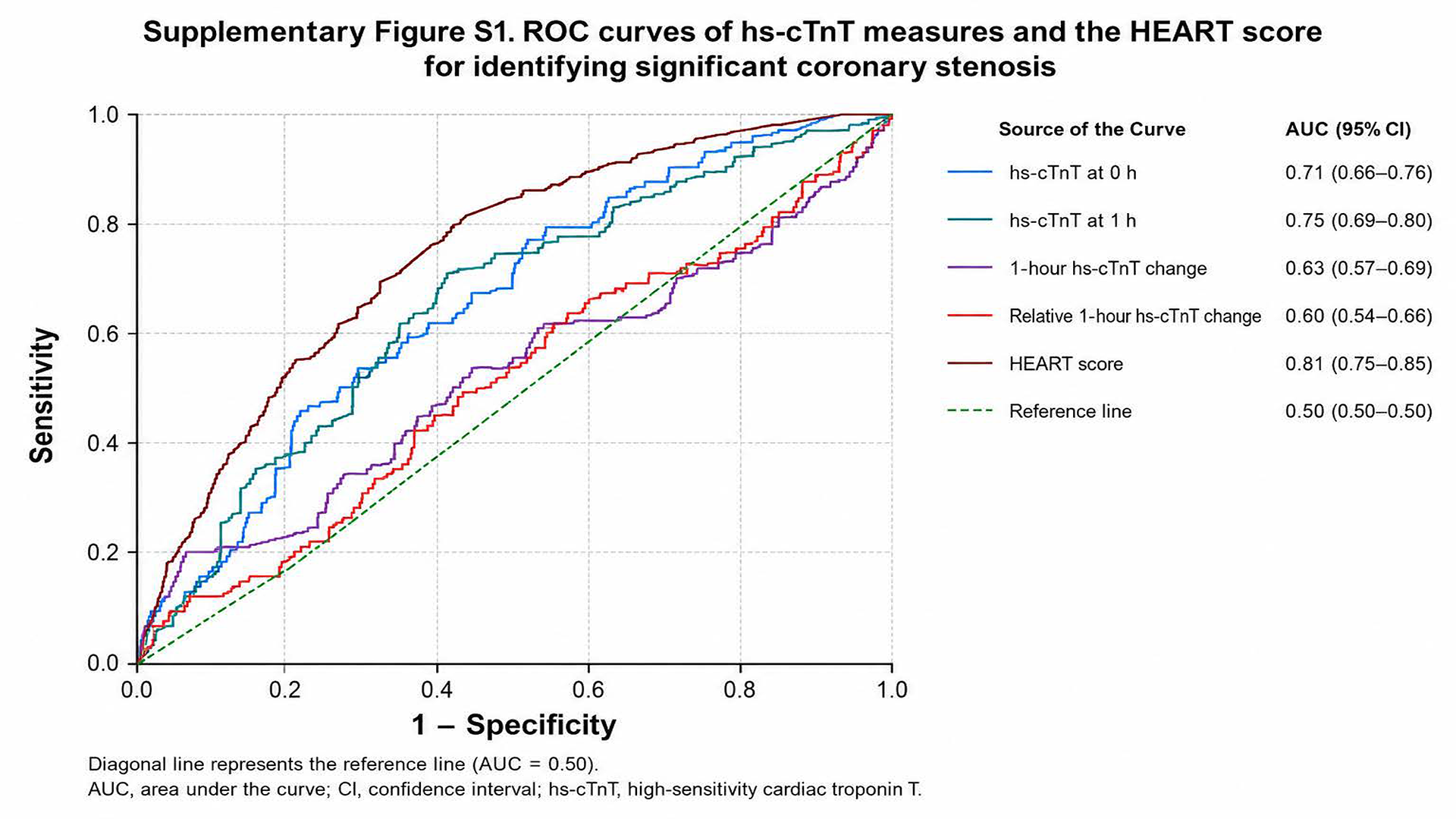

Supplement: online supplemental figure 1 [file openhrt-13-2-s001.tif]
