# Supplementary material for: Coronary angiography findings in emergency department chest pain patients undergoing angiography despite hs-cTnT-based early rule-out angiography after hs-cTnT rule-out in ED chest pain
Source: Open Heart. 2026 Jul 9;13(2):e004186. doi: 10.1136/openhrt-2026-004186 (PMC13358279; doi:10.1136/openhrt-2026-004186)
Supplement: online supplemental table 1 [file openhrt-13-2-s002.docx]

**Table S1. Bonferroni-adjusted pairwise comparisons according to clinical management pathway**

| **Variable** | **Pairwise comparison** | **Adjusted p value** |
| --- | --- | --- |
| Age, years | Discharged vs urgent CAG | <0.001 |
|  | Discharged vs elective CAG | <0.001 |
| 1-hour change in hs-cTnT, ng/L | Urgent CAG vs elective CAG | 0.010 |
|  | Urgent CAG vs discharged | <0.001 |
| Relative 1-hour change  in hs-cTnT, % | Urgent CAG vs elective CAG | 0.006 |
|  | Urgent CAG vs discharged | <0.001 |
| HEART score | Discharged vs elective CAG | <0.001 |
|  | Discharged vs urgent CAG | <0.001 |
| HEART history component | Elective CAG vs urgent CAG | <0.001 |
|  | Discharged vs urgent CAG | <0.001 |
| HEART ECG component | Urgent CAG vs elective CAG | <0.001 |
|  | Discharged vs elective CAG | <0.001 |
| HEART age component | Discharged vs urgent CAG | <0.001 |
| HEART risk-factor component | Discharged vs elective CAG | <0.001 |
|  | Discharged vs urgent CAG | <0.001 |
|  | Elective CAG vs urgent CAG | 0.001 |

Only statistically significant pairwise comparisons are shown. Pairwise post-hoc comparisons were performed following the Kruskal–Wallis test, and p values were adjusted using the Bonferroni correction.CAG, coronary angiography; ECG, electrocardiography; hs-cTnT, high-sensitivity cardiac troponin T.
