# Supplementary material for: Coronary angiography findings in emergency department chest pain patients undergoing angiography despite hs-cTnT-based early rule-out angiography after hs-cTnT rule-out in ED chest pain
Source: Open Heart. 2026 Jul 9;13(2):e004186. doi: 10.1136/openhrt-2026-004186 (PMC13358279; doi:10.1136/openhrt-2026-004186)
Supplement: online supplemental table 2 [file openhrt-13-2-s003.docx]

**Table S2. Bonferroni-adjusted pairwise comparisons of hs-cTnT parameters according to angiographic outcome**

| **Variable** | **Pairwise comparison** | **Adjusted p value** |
| --- | --- | --- |
| hs-cTnT at 0 h | Group 1 vs Group 2 | 0.002 |
|  | Group 1 vs Group 3 | <0.001 |
|  | Group 2 vs Group 3 | <0.001 |
| hs-cTnT at 1 h | Group 1 vs Group 2 | <0.001 |
|  | Group 1 vs Group 3 | <0.001 |
|  | Group 2 vs Group 3 | 0.011 |
| Absolute Δ hs-cTnT | Group 2 vs Group 3 | 0.026 |
| Relative Δ hs-cTnT, % | Group 2 vs Group 3 | 0.003 |

Only statistically significant pairwise comparisons are shown. Pairwise post-hoc comparisons were performed following the Kruskal–Wallis test, and p values were adjusted using the Bonferroni correction. Group 1: significant coronary stenosis requiring intervention; Group 2: intermediate coronary stenosis managed medically; Group 3: normal coronary arteries.hs-cTnT, high-sensitivity cardiac troponin T.
