# Supplementary material for: Coronary angiography findings in emergency department chest pain patients undergoing angiography despite hs-cTnT-based early rule-out angiography after hs-cTnT rule-out in ED chest pain
Source: Open Heart. 2026 Jul 9;13(2):e004186. doi: 10.1136/openhrt-2026-004186 (PMC13358279; doi:10.1136/openhrt-2026-004186)
Supplement: online supplemental table 3 [file openhrt-13-2-s004.docx]

**Table S3. Bonferroni-adjusted pairwise comparisons of HEART score and its components according to angiographic outcome**

| **Variable** | **Pairwise comparison** | **Adjusted p value** |
| --- | --- | --- |
| HEART score | Group 1 vs Group 2 | <0.001 |
|  | Group 1 vs Group 3 | <0.001 |
|  | Group 2 vs Group 3 | <0.001 |
| History component | Group 1 vs Group 2 | 0.001 |
|  | Group 1 vs Group 3 | <0.001 |
| ECG component | Group 1 vs Group 3 | 0.004 |
| Age component | Group 1 vs Group 3 | <0.001 |
|  | Group 2 vs Group 3 | <0.001 |
| Risk-factor component | Group 1 vs Group 2 | <0.001 |
|  | Group 1 vs Group 3 | <0.001 |
|  | Group 2 vs Group 3 | 0.002 |

Only statistically significant pairwise comparisons are shown. Pairwise post-hoc comparisons were performed following the Kruskal–Wallis test, and p values were adjusted using the Bonferroni correction. Group 1: significant coronary stenosis; Group 2: intermediate coronary stenosis; Group 3: Non-obstructive coronary artery disease; ECG, electrocardiography; HEART, History, ECG, Age, Risk factors, and Troponin.
