# Supplementary material for: Coronary angiography findings in emergency department chest pain patients undergoing angiography despite hs-cTnT-based early rule-out angiography after hs-cTnT rule-out in ED chest pain
Source: Open Heart. 2026 Jul 9;13(2):e004186. doi: 10.1136/openhrt-2026-004186 (PMC13358279; doi:10.1136/openhrt-2026-004186)
Supplement: online supplemental table 4 [file openhrt-13-2-s005.docx]

**Table S4. ROC performance of hs-cTnT measures and the HEART score for identifying significant coronary stenosis**

| **Variable AUC** | |  | **95% CI** | **p value** | **Optimal cut-off** | **Sensitivity** | **Specificity** |
| --- | --- | --- | --- | --- | --- | --- | --- |
| hs-cTnT at 0 h | 0.648 | | 0.588–0.708 | <0.001 | ≥8.18 ng/L | 45.9% | 78.3% |
| hs-cTnT at 1 h | 0.664 | | 0.605–0.722 | <0.001 | ≥6.35 ng/L | 75.2% | 56.2% |
| 1-hour hs-cTnT change | 0.527 | | 0.459–0.595 | 0.408 | ≥1.40 ng/L | 18.3% | 94.4% |
| Relative 1-hour hs-cTnT change | 0.512 | | 0.446–0.578 | 0.718 | ≥5.48% | 51.4% | 56.9% |
| HEART score | 0.697 | | 0.640–0.754 | <0.001 | ≥4 | 78.9% | 51.3% |

The positive outcome was significant coronary stenosis (Group 1), whereas intermediate stenosis and non-obstructive coronary artery disease (Groups 2 and 3) constituted the negative outcome. Optimal cut-off values were determined using the Youden index. AUC, area under the receiver operating characteristic curve; CI, confidence interval; hs-cTnT, high-sensitivity cardiac troponin T.
