# Supplementary material for: Coronary angiography findings in emergency department chest pain patients undergoing angiography despite hs-cTnT-based early rule-out angiography after hs-cTnT rule-out in ED chest pain
Source: Open Heart. 2026 Jul 9;13(2):e004186. doi: 10.1136/openhrt-2026-004186 (PMC13358279; doi:10.1136/openhrt-2026-004186)
Supplement: online supplemental table 5 [file openhrt-13-2-s006.docx]

**Table S5. Documented procedural and angiographic management details among patients with significant coronary stenosis and available intervention-site data**

| **Documented procedure or management finding** | **n (%)** |
| --- | --- |
| LAD PCI | 20 (21.5) |
| LCx PCI | 18 (19.4) |
| RCA PCI | 18 (19.4) |
| CABG referral or decision | 13 (14.0) |
| LAD + LCx PCI | 4 (4.3) |
| LCx balloon angioplasty | 3 (3.2) |
| RCA chronic total occlusion | 3 (3.2) |
| LCx + RCA PCI | 3 (3.2) |
| LAD balloon angioplasty | 2 (2.2) |
| Balloon angioplasty of previously implanted stents | 1 (1.1) |
| IMA PCI | 1 (1.1) |
| Diagonal branch balloon angioplasty | 1 (1.1) |
| PCI, vessel unspecified | 1 (1.1) |
| OM PCI | 1 (1.1) |
| RCA balloon angioplasty | 1 (1.1) |
| D1 PCI | 1 (1.1) |
| LMCA + LAD + LCx + RCA PCI | 1 (1.1) |
| CABG after a previous operation | 1 (1.1) |
| **Total** | **93 (100.0)** |

Data are presented for 93 of 109 patients with significant coronary stenosis for whom intervention-site or referral information was available. The two synonymous entries “LCx + RCA PCI” and “RCA + LCx PCI” were combined. Categories reflect the documented procedure, referral decision, or angiographic management finding and do not constitute standardized culprit-lesion adjudication. PCI, percutaneous coronary intervention; CABG, coronary artery bypass grafting; LAD, left anterior descending artery; LCx, left circumflex artery; RCA, right coronary artery; CTO, chronic total occlusion; OM, obtuse marginal branch; D1, first diagonal branch; LMCA, left main coronary artery; IMA, internal mammary artery.
